# Visual Attention in Joint Attention Bids: A Comparison Between Toddlers with Autism Spectrum Disorder and Typically Developing Toddlers

**DOI:** 10.1007/s10803-023-06224-y

**Published:** 2024-02-12

**Authors:** Selda Ozdemir, Isik Akin-Bulbul, Erol Yildiz

**Affiliations:** 1 Department of Special Education, Education Faculty, Hacettepe University, Beytepe, Ankara, Turkey; 2 Department of Special Education, Gazi Education Faculty, Gazi University, Teknikokullar, Ankara, Turkey; 3 Department of Special Education, Hacettepe University, Beytepe, Ankara, Turkey

**Keywords:** Autism, Joint attention, Visual attention, Eye tracking, Eye movement, Intelligence, Language, Gender

## Abstract

Impairments in joint attention are considered core symptoms of autism spectrum disorder (ASD) and are crucial for early assessment and intervention. However, existing information about visual attention during joint attention and its relation to developmental functioning is limited. The current study investigated the visual attention differences between toddlers with ASD and typically developing (TD) toddlers during single and multiple joint attention cue conditions. This study further examined whether visual attention in joint attention Area of Interests (AOIs) is related to cognitive, language, and motor development in participants. Participants included 56 toddlers with ASD and 56 TD toddlers, aged 18–36 months. A passive viewing paradigm was used to assess participants’ visual attention across four different joint attention conditions. Study results showed that toddlers with ASD displayed limited visual attention towards joint attention AOIs, Target, Face, and Target and Face AOIs, in all conditions. As the joint attention cues increased from Condition 1 to Condition 4, toddlers with ASD showed increased visual attention directed towards the Target AOI over the Face AOI. Moreover, increased visual attention on the joint attention AOIs was associated with improved cognitive and language development in toddlers with ASD. This research broadens our understanding of the positive relationships between visual attention to joint attention AOIs and cognitive as well as language development in toddlers with ASD, suggesting that early interventions targeting joint attention may offer promising pathways for treatment.

Joint attention is defined as a triadic organization of visual attention and communication, including attending to both communication partner and referent (an object, event, or person) during shared interactions (Mundy & Newel, 2007). The behavioral dimension of joint attention involves the integration of eye gaze, prelinguistic gesture, and vocalization in the transition to first words (Braddock & Brady, 2016). Numerous studies have shown that early joint attention impairments in the first 2 years of life are the primary indicators of social communication difficulties in young children with autism spectrum disorder (ASD) (Charman, 2003) and a strong predictor of a later ASD diagnosis (Landa et al., 2007; Rozga et al., 2011; Sullivan et al., 2007).

Joint attention in young children can be classified into two primary categories depending on whether an infant responds to others’ joint attention bids (RJA) or initiating joint attention (IJA). Researchers showed that both joint attention skills are impaired in young children with ASD (Mundy, 2017; Mundy et al., 1986), and the impairments range from qualitative differences to a complete absence of joint attention (Braddock & Brady, 2016). In a study, Yoder et al. (2009) examined joint attention impairment in at-risk infants. Researchers found that early difficulties in RJA and IJA, including both requesting and sharing, predicted later ASD diagnosis. Sullivan et al. (2007) further explored the relationship between RJA and ASD diagnosis. The authors found that failure to respond to gaze and point cues at 14 and 24 months predicted ASD diagnosis at 30–36 months. Later research by Bedford et al. (2012) revealed similar results and showed that 13-month-old babies later diagnosed with ASD were poorer at orienting to the target in response to gaze cues only. Recent evidence also showed that impairments in RJA at 14 and 15 months remained significant predictors of ASD diagnosis (Rozga et al., 2011).

Despite the well-established evidence of joint attention impairments in young children with ASD (Ibanez et al., 2013; Mundy, 1995; Mundy et al., 2000), there have been studies showing no significant group differences in RJA between children with ASD and TD comparisons (e.g., Goldberg et al., 2005; Yirmiya et al., 2006). Such findings might be attributable to differences in the heterogeneity of the condition, differences in participants’ age and clinical characteristics, the wide variety of the research paradigms (Setien-Ramos et al., 2022), and joint attention cue-types, with single and multiple cues used in the experiments (e.g., calling the child’s name, pointing, and looking at an object). For example, Presmanes et al. (2007) explored ten different combinations of verbal and nonverbal joint attention cues with younger siblings of children with ASD (SIBS-ASD) and siblings of TD children (SIBS-TD). Authors reported that moderately redundant cues were most difficult for SIBS-ASD; however, adding a point cue significantly improved RJA for SIBS-ASD.

Research on visual attention has expanded significantly over the past few decades, mainly due to the availability of new eye-tracking technology. This technology enables researchers to examine eye movements as markers of visual attention and perception. This line of research has generated important insights into identifying mechanisms underlying social impairments in individuals with ASD (Setien-Ramos et al., 2022). Experiments investigating the eye movements of toddlers with ASD showed that these toddlers are less likely than TD children to focus on faces (Chawarska et al., 2010; Jones et al., 2008; Ozdemir et al., 2018; Shic et al., 2011). Moreover, various studies indicated reduced preferential attention to social stimuli compared to non-social stimuli across diverse age groups (Campbell et al., 2014; Chawarska et al., 2012; Crawford et al., 2016; Falck-Ytter et al., 2013; Klin et al., 2009; Ozdemir et al., 2022). Numerous studies suggest that ASD is associated with increased visual attention towards objects (Moriuchi et al., 2016; Sabatino, 2013; Sasson & Touchstone, 2014; Turner-Brown et al., 2011). Additionally, Baranek et al. (2018) reported that decreased visual attention disengagement in high-risk infants at 12–15 months correlated with diminished social orienting at 20–24 months.

A recent meta-analysis by Chita-Tegmark (2016) found that children with ASD paid less attention to social cues than their peers without ASD, with an overall effect size of 0.55 across 38 papers. Other investigations showed that children with ASD spend more time on outside Area of Interest (AOI) (background of the stimuli) than control groups both on static (Chawarska & Shic, 2009; Riby & Hancock, 2008) and dynamic stimuli (Akin-Bulbul & Ozdemir, 2023; Nakano et al., 2010). Thorup et al. (2016) investigated gaze direction and discovered that 10 months old low-risk newborns were able to follow gaze in both the Gaze only and Gaze and Head conditions, while high-risk infants were only able to do so in the Gaze and Head condition. According to the authors, head movements had a greater impact on high-risk infants’ gaze-following responses than they did on low-risk newborns. Similarly, Vivanti et al. (2017) showed that children with ASD displayed difficulty following gaze and lacked preferential attention to social versus non-social stimuli. Moreover, Gillespie-Lynch et al. (2013) found that children with ASD exhibited impaired gaze following, while another study showed that infants’ ability to follow gaze improved when verbal cues accompanied pointing (Leekam et al., 1998).

Recently, eye-tracking methodology was used during live interactions to examine the impact of head movement on infants’ gaze following ability (Nyström et al., 2019; Thorup et al., 2016). One study reported that infants at risk of ASD were less likely to follow gaze in the Eyes-Only condition than in the Eyes/Head condition (Thorup et al., 2016). In Belen et al.’s (2023) study, researchers found that children diagnosed with ASD were less likely to follow gaze cues compared to TD children. Children with ASD had lower accuracy in following gaze when they could only see eye gaze information, compared to when they could observe both eye gaze and head movement. However, this pattern was statistically stronger in TD children.

Despite the substantial evidence base, literature on visual attention differences in children with ASD presents contradictory findings. These discrepancies may stem from the clinical characteristics of children with ASD, properties of eye-tracking stimuli such as dynamic videos versus static images (Cilia et al., 2019; Ozdemir et al., 2018), the emotional content of the stimuli (Franchini et al., 2017), initiation of direct gaze (Nyström et al., 2017), and the visibility of the joint attention targets (Congiu et al., 2016). For example, Billeci et al. (2016) reported no differences in the RJA task between ASD and TD children. Similarly, Cilia et al. (2019) found no visual attention differences in static and dynamic joint attention tasks between children with ASD and TD children. Both groups spent more time looking at the Face and Target AOIs. While no group differences were identified, the mean age of the participants (M = 90.43 months for ASD participants) was higher than that typically observed in joint attention studies.

Furthermore, previous behavioral studies have investigated associations between joint attention and social, cognitive, adaptive, and language skills in children with ASD (Bottema-Beutel, 2016; Harrison et al., 2016; Mundy et al., 1994; Sano et al., 2021). The link between visual attention in joint attention and language development is particularly significant, with several publications specifically addressing this relationship (Falck-Ytter et al., 2012; Leekam et al., 1998). Failing to engage in joint attention can adversely affect a child’s development in various domains, including language development, where visual attention is essential for linking words with their corresponding referents. However, the connection between joint attention and cognitive skills remains a subject of debate. Some research, such as the studies by Harrison et al. (2016) and Mundy et al. (1994) provided limited evidence regarding the correlation between intelligence and joint attention in children with ASD. Mundy et al. (1994) found that lower intelligence was related to greater impairment in low-level joint attention in children with ASD. However, these findings cannot be generalized to children with ASD who have higher intelligence and lower autism symptoms. Building on this line of research, a recent study by Sano et al. (2021) showed that lower intelligence is linked with joint attention impairment in children with ASD, including participants with higher intelligence and potentially milder autistic symptoms. Yet, due to the complexity of the research variables, the evidence linking joint attention to cognitive development in children with ASD remains inconclusive.

Moreover, there is increasing interest in exploring gender differences in the development and clinical profiles of females on the autism spectrum. Harrop et al. (2019) found significant differences in visual attention patterns between males and females with ASD. Specifically, females with ASD displayed more attention to faces in socially lean conditions compared to their male peers. Recent studies reported condition x sex interactions, indicating that both children with ASD and TD children tend to focus on images that align with interests typically associated with their biological sex (Harrop et al., 2018, 2019, 2020).

Visual attention is one of the key mechanisms of perception that allows young children to efficiently select the most relevant information while avoiding information overload. Research shows that the development of social cognitive processes involved in understanding joint attention cues contributes to the maturation of children’s visual-spatial ability in encoding locations (Mundy et al., 2000). Visual attention, therefore, can be a clear indicator of a young child’s emerging capacities for joint attention. While reduced visual attention to social stimuli has been widely reported in individuals with ASD, it is unclear how this reduced attentional bias manifests during joint attention, implicit, and explicit cues. To date, joint attention impairments in ASD have been mainly assessed by monitoring the gaze line, with or without head orientation, as seen in previous studies (Bedford et al., 2012; Falck-Ytter et al., 2012, 2015; Riby & Doherty, 2009). However, many studies often lack the necessary sensitivity to identify the visual attention differences in joint attention cues, such as pointing gestures and verbal cue like “Look!”.

Overall, previous research has failed to establish a strong link between joint attention impairment and visual attention of young children with ASD during implicit and explicit joint attention cues. Therefore, in this study, we examined the visual attention of toddlers with ASD and TD toddlers under single and multiple joint attention cue conditions. Four different conditions from implicit to explicit joint attention bids were designed to examine the attention cuing paradigm: Eye Gaze Condition-1, Eye Gaze and Head Turning Condition-2, Eye Gaze, Head Turning, and Pointing Condition-3, and Eye Gaze, Head Turning, Pointing, and “Look!” Condition-4. We expected reduced visual attention into joint attention AOIs during implicit joint attention cue conditions such as Eye Gaze Condition-1 and increased visual attention during the most explicit joint attention condition, including Eye Gaze, Head Turning, Pointing, and “Look!” Condition-4.

We predict that, in comparison to TD children, children with ASD will show attention inhibition difficulties across joint attention cue conditions if visual attention inhibition is central to understanding joint attention impairment. In addition, the more ambiguous a joint attention task (e.g., Eye Gaze Condition-1), the more effort it will take the toddlers with ASD to attend the joint attention AOIs. In contrast, an explicit joint attention condition will require less demands for planning and executive regulation of visual attention, hence the greater likelihood that young children with ASD will perform better than implicit joint attention cue conditions. Children with ASD may have difficulty attending to social cues due to an inability to inhibit shifts in visual attention towards distracting stimuli.

Although the behaviors underlying the construct of joint attention impairments in toddlers with ASD are well documented, less is known about the developmental relationships that contribute to joint attention impairments. Visual attention is a central part of social information processing and joint attention difficulties affect a child’s learning opportunities dramatically. Thus, in the current study, we investigated the correlations between visual attention and various clinical assessments, including cognitive, language, motor, and gender variables. The current study builds on the emerging autism and visual attention literature that examines the link between developmental variables and visual attention across joint attention conditions.

## Method

### Participants

This study was conducted by the declaration of Helsinki and was approved by the ethics committee of Gazi University and the Scientific and Technological Research Council of Turkey (Grant #115K459). Parents provided written informed consent on behalf of their child for participation in the study. 56 toddlers diagnosed with ASD, with a mean age of 33.84 months (SD: 6.86), were recruited from a university-based research center cohort, along with 56 TD toddlers with a mean age of 24.75 months (SD: 4.05). Outliers were excluded from the final analysis, resulting in 18 study participants being excluded (9 from each group). Webb et al. (2020) recommend assessing the distribution of variances in biomarker data to determine acceptable variability.

All participants met the following study inclusion criteria; age between 18 and 36 months, absence of seizure disorder or known genetic disorder, and absence of a hearing or visual impairment uncorrectable with glasses. Toddlers with ASD held a primary diagnosis of ASD from a child psychiatrist based on the DSM-V (American Psychiatric Association, 2013) criteria for autism spectrum disorder in state hospitals. Children’s diagnoses were confirmed via clinical assessments conducted by the research team specializing in the early diagnosis of ASD. Clinical assessments included a psychiatric interview and behavioral observations to assess the social communication impairments and repetitive and stereotyped behaviors. The TD control group was recruited from the local community. Participants of the control group exhibited no symptoms of developmental, behavioral, or emotional disorders. Additionally, they had no history of ASD in any 1st, and 2nd-degree relatives, and none of their older or younger siblings displayed any symptoms of developmental, behavioral, or emotional disorders. The ASD and TD groups were matched based on their cognitive developmental age, as shown in Table 1. The children’s cognitive skills were measured on Bayley Scales of Infant and Toddler Development, Third Edition (BAYLEY-III).

**Table 1:** Demographic and clinical features of the participants

Variable	ASD group (n = 56)			TD group (n = 56)			t-test score (p-value)
	Mean	SD	Range	Mean	SD	Range	
Age in months	33.84	6.86	22	24.75	4.05	19	− 8.54 (.00**)
Male/female	45/11	–	–	30/26	–	–	–
Bayley cog.	23.23	6.49	27	24.52	4.56	21	1.21 (.23)
Composite lang.	72.21	15.54	54	101.93	9.28	42	12.29 (.00**)
Expressive lang.	20.93	10.13	33	24.70	8.05	29	2.18 (.03*)
Receptive lang.	20.57	8.92	30	26.21	7.14	31	3.70 (.00**)
Composite motor	66.63	19.55	73	100.52	9.52	42	11.67 (.00**)
Fine motor	24.77	7.91	42	25.45	6.45	25	.50 (.62)
Gross motor	25.50	9.84	55	25.29	4.79	19	− .015 (.88)
M-Chat class							
No risk	–			56			–
Medium risk	4			–			–
High risk	52			–			–

*TD* typically developing, *ASD* autism spectrum disorder, *M-Chat* Modified Checklist for Autism in Toddlers

*p < .05, **p < .01

In addition, the Modified Checklist for Autism in Toddlers Revised Form (M-CHAT-R/F, Robins et al., 2013) was used to report the early ASD risk profile. While recent studies have highlighted the low to moderate accuracy of developmental screeners, including the M-CHAT-R/F, in identifying ASD in young children (Guthrie et al., 2019; Yuen et al., 2018), we used the M-CHAT-R/F because it was the only available standardized screening instrument in the native language of toddlers with ASD (e.g., Autism Diagnostic Observation Schedule (ADOS) was not available). The demographic and clinical features of the study participants are presented in Table 1.

### Stimuli

**Fig. 1:**
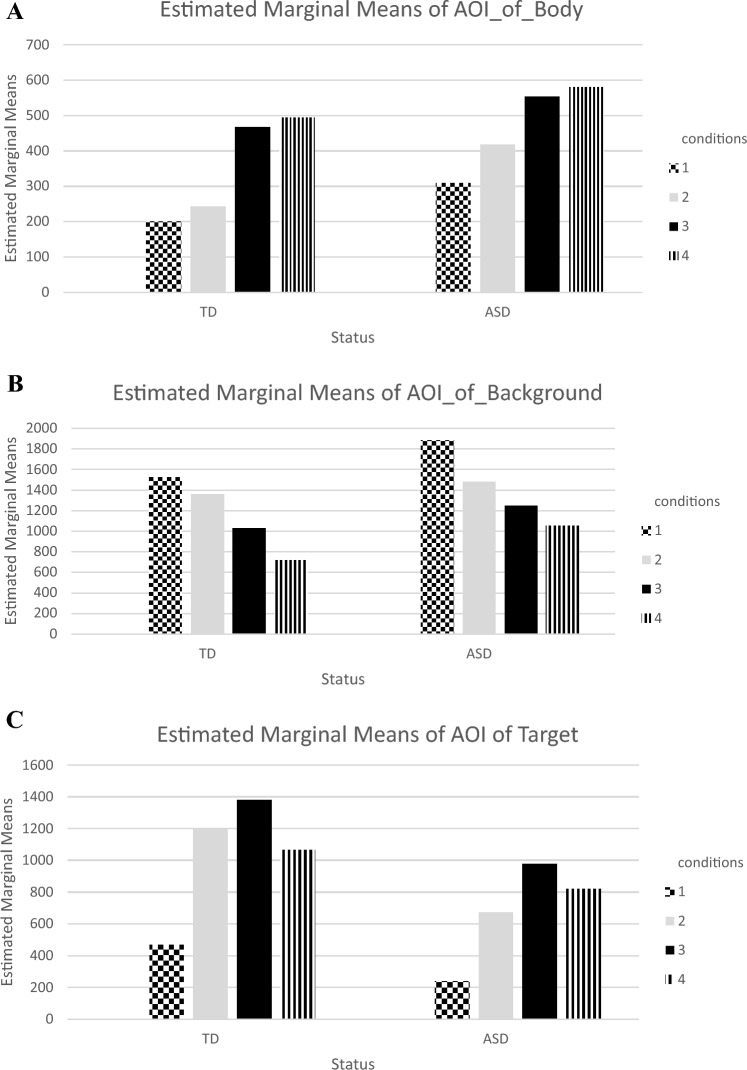

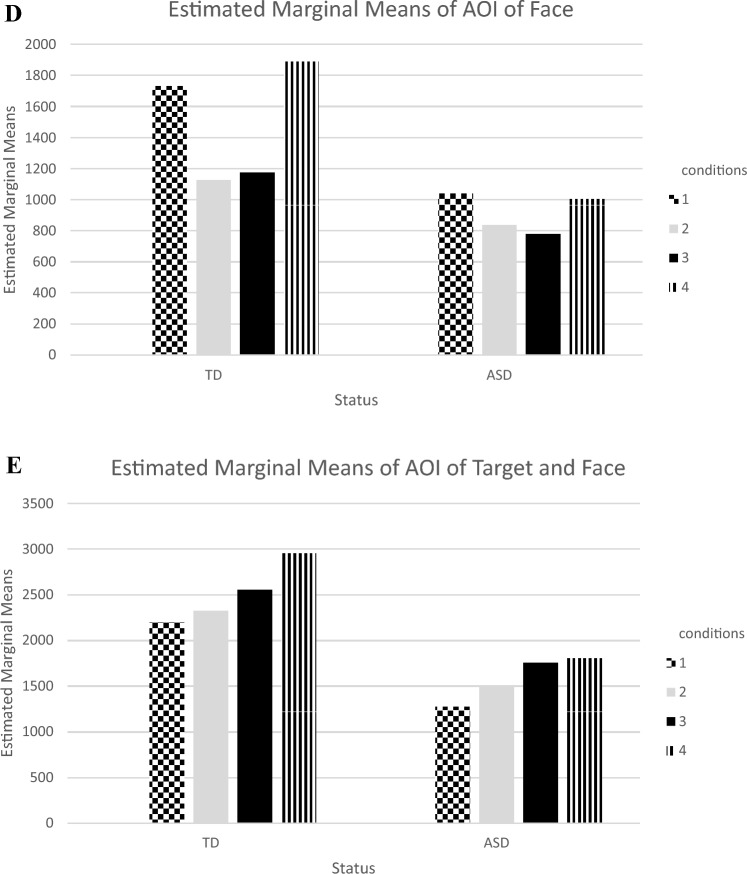
**A** Net dwell time of toddlers with ASD and TD toddlers in joint attention cue conditions: AOI of body. **B** Net dwell time of toddlers with ASD and TD toddlers in joint attention cue conditions: AOI of background. **C** Net dwell time of toddlers with ASD and TD toddlers in joint attention cue conditions: AOI of target. **D** Net dwell time of toddlers with ASD and TD toddlers in joint attention cue conditions: AOI of face. **E** net dwell time of toddlers with ASD and TD toddlers in joint attention cue conditions: AOI of target and face

Eye movements of the participants were measured using a passive viewing paradigm in which the nature of the task does not constrain attention. The experiment consisted of four joint attention conditions, with each condition presented two times including both sides of the screen, right and left. In the Eye-Gaze Condition-1, the actress looked directly at the camera and then directed her gaze to a target toy on the table (Condition 1). In the Eye-Gaze and Head-Turning Condition-2, the actress looked directly at the camera, then directed her gaze to a target toy on the table and turned her head along with her eyes (Condition 2). In the Eye-Gaze, Head-Turning, and Pointing Condition-3, the actress looked directly at the camera, then directed her gaze to a target toy on the table, turned her head along with her eyes, and extended her arm and index finger to point to the target toy (Condition 3). Finally, in the Eye-Gaze, Head-Turning, Pointing, and “Look!” Condition-4, the actress looked directly at the camera, and then directed her gaze to a target toy on the table, turned her head along with her eyes, extended her arm and index finger to point the target toy, and gave the verbal cue, “Look!” (Condition 4). The stimuli consisted of a total of eighty seconds video (Four joint attention conditions and eight trials, with each condition, presented two times on the right and left sides of the screen) of an actress filmed in a setting containing toys and a table (See Fig. 1). Joint attention conditions were developed based on the ADOS-II (Lord et al., 2012) assessment. This approach was chosen because ADOS is widely accepted as a gold standard diagnostic tool for ASD. Previous studies have been conducted to assess joint attention using ADOS-derived joint attention tasks, including studies by Belen et al., 2023; Billeci et al., 2016; Leekam et al., 1998; Presmanes et al., 2007.

The current study involved showing the participants eight videos of an actress seated behind a table that had toys on each side of the actress. The target objects were colorful, attractive, and easily recognizable toys for young children (e.g., books, toy cars, blocks, etc.). In each condition, the toddlers watched the joint attention cues at objects in the periphery. The screen’s left and right sides were used equally in each condition to control participants’ possible left and right-side bias.

Each stimulus consisted of two 8-s videos, and before each stimulus, there was a 2-s red fixation dot video segment so that the participants’ eye movements could be centered. In the first stage, the actress looked directly at the camera for 2-s seconds with a neutral facial expression. Following this, the actress initiated joint attention (Eye-Gaze, Eye-Gaze + Head Turning, Eye-Gaze + Head Turning + Pointing, and Eye-Gaze + Head Turning + Pointing + Look Conditions) for 4-s in each condition. Lastly, the actress shifted her gaze back to the camera for 2-s. Each video representing the conditions lasted 10-s while the participants watched a video recording for a total of 80 s. We automatically excluded visual attention data related to the 2-s (red fixation dot) segments between videos, because we used only AOIs to extract children’s visual attention data.

### Apparatus and Procedure

Eye-tracking data were collected in the passive viewing paradigm. The experiment was conducted in an eye-tracking laboratory specially designed for eye-tracking data collection, located in a university research center. Participants were tested individually in the eye tracking laboratory of the research center. The laboratory had two rooms separated by a one-way glass window for researcher and caregiver observation. In the inner room, the participant and his/her parent were seated in a comfortable chair at a viewing distance of approximately 60 cm from the monitor. The researcher was in the inner room to monitor the calibration and research process. The eye tracking task was initiated when at least four points were marked as correctly calibrated for both eyes, and participants were told that they were going to watch the videos.

Study data were recorded and stored using SMI Experiment Center™ 3.6 software and database. Stimuli were displayed on a 17-inch monitor with a 250-Hz refresh rate. Eye movements of the participants were recorded using an SMI eye-tracking system. BeGaze 3.6 software-controlled response, including data calibration, recalibration, Area of Interest (AOI), and blink detection. The screen resolution was set to 1680 × 1050 pixels. A five-point calibration procedure was performed before recording using an animated image presented at known X–Y coordinates. A crying carton was used as a calibration image to catch the children’s attention on the screen. The calibration procedure required gaze following on an image of a crying carton, starting with the center of the screen, and moving across the four corners of the screen. The calibration process was repeated for each child until the deviation for the x and y components was below 1°. Trials were excluded if there was no fixation recorded on the Face AOI during the attention-getter and/or the smiling phase, as this indicated that the child was not reliable. For the analysis, each participant needed to have at least one valid trial (which accounts for 25% or more) in each condition. 65 children with ASD and 65 TD children met this criterion for the initial conditions.

Subsequently, each child was presented with the eight joint attention video clips described in the stimulus section. Net dwell time was the dependent variable of the study. Net dwell time reflects the sum of sample durations for all gaze data samples that hit the AOI. The visual scene was divided into four AOIs: 1: Face, 2: Body, 3: Target, and 4: Background AOIs. For research purposes, a fifth AOI was extracted and calculated from two AOIs, the Target AOI and Face AOI, named the Target and Face AOI as the joint attention visual area includes both the Face and Target AOIs. Lastly, nonoverlapping AOIs were defined using a manual drawing in SMI software.

## Results

### Between Group Analysis on Joint Attention Cue Conditions

The present study investigated differences in visual attention across five AOIs (1: Face, 2: Body, 3: Target, 4: Background, 5: Face and Target AOIs) between toddlers with ASD and TD toddlers in four joint attention conditions: Eye-Gaze Condition-1, Eye-Gaze and Head-Turning Condition-2, Eye-Gaze, Head-Turning, and Pointing Condition-3, and Eye-Gaze, Head-Turning, Pointing, and “Look!” Condition-4. Details can be seen in Fig. 1. After the data cleaning process, net dwell time of both study groups was compared using a MANOVA test. Recognizing that data cleaning is a prerequisite for many multivariate analyses, we processed the raw data with rigorous data cleaning steps. This process involved detecting missing values, outliers, and checking out normal distribution. Initially, we inspected the dataset for any missing values and confirmed that there were no missing values. Following this process, we determined that five data sets were not normally distributed, as evidenced by positive skewness values, indicating a right-skewed distribution.

Therefore, we identified 18 outliers and removed them from the dataset. After eliminating these extreme outliers, we reached the optimal skewness values within the range of ± 1.5 (Tabachnick & Fidell, 2013). Webb et al. (2020) suggest that biomarker values should have appropriate distributional properties. These include a skew value less than 2 (with checking for values not equal to 0), a kurtosis value less than 3 (with checking for values not equal to 0), and not having floor/ceiling effects or zero inflation. The presence of distributional issues in toddlers with ASD could represent an important marker, not necessarily discrediting data. Such outliers might indicate a distinct underlying pathophysiological impairment. Finally, we estimated effect sizes using partial eta squared. Based on Cohen (1988)’s index, effect sizes were interpreted as small, medium, and large, corresponding to the values of n^2^ = .0099, .0588, and .1379, respectively (Richardson, 2011).

#### Eye-Gaze Condition-1

In the Eye-Gaze Condition-1, toddlers with ASD exhibited significantly lower net dwell time than the TD group in the Target AOI, the Face AOI, and the Target and Face AOI (F_t_ = 12.65, p = .01; F_f_ = 28.65, p = .00; F_tf_ = 38.80, p = .00). In contrast, ASD toddlers had significantly higher net dwell time than the TD toddlers in the Body AOI and the Background AOI (F_b_ = 8.72, p = .01; F_bc_ = 7.60, p = .01). The effect sizes for these differences (n^2^ = .10, .21, .26, .07, .07) are characterized as medium, large, and medium effects respectively (Cohen, 1988). Table 2 shows the effect sizes derived from the analysis.

**Table 2:** MANOVA results in net dwell time of toddlers with ASD and TD toddlers

	Groups	N	Mean	SD	Range	F	p	Partial eta squared
Eye gaze								
Body	TD	56	200.29	124.85	553.90	8.72	.01*	.07
	ASD	56	327.48	297.21	1214.15			
Background	TD	56	1519.21	741.71	2966.95	7.60	.01*	.07
	ASD	56	1987.22	1031.59	1987.22			
Target	TD	56	467.90	371.91	1532.00	12.65	.01*	.10
	ASD	56	248.69	272.93	1103.90			
Face	TD	56	1727.48	914.92	3594.10	28.65	.00**	.21
	ASD	56	877.85	757.46	2668.20			
Target and face	TD	56	2195.39	996.84	3818.30	38.80	.00**	.26
	ASD	56	1126.55	809.33	2963.65			
Eye gaze, head-turning								
Body	TD	56	245.52	159.19	690.30	14.20	.00**	.11
	ASD	56	428.92	327.57	1253.65			
Background	TD	56	1351.60	632.52	2461.75	3.72	.06	.03
	ASD	56	1642.91	936.93	1642.91			
Target	TD	56	1194.95	672.84	2869.70	22.45	.00**	.17
	ASD	56	666.34	494.16	1865.25			
Face	TD	56	1130.13	755.13	3209.85	12.80	.01*	.10
	ASD	56	666.21	609.60	2323.85			
Target and face	TD	56	2325.08	933.66	3717.60	35.62	.00**	.25
	ASD	56	1330.76	826.13	3243.10			
Eye gaze, head-turning, pointing								
Body	TD	56	466.17	345.66	1627.95	2.63	.11	.02
	ASD	56	607.33	552.68	607.33			
Background	TD	56	1029.29	552.73	2393.75	5.72	.02*	.05
	ASD	56	1352.20	846.04	3703.90			
Target	TD	56	1389.01	643.72	2419.70	5.07	.03*	.04
	ASD	56	1062.63	873.42	3135.80			
Face	TD	56	1171.01	643.13	3325.95	20.08	.00**	.15
	ASD	56	638.09	615.33	2047.90			
Target and face	TD	56	2560.03	772.56	2891.25	21.36	.00**	.16
	ASD	56	1700.60	1157.45	3827.75			
Eye gaze, head-turning pointing, and “Look!”								
Body	TD	56	493.77	373.59	1639.70	2.10	.15	.02
	ASD	56	635.87	631.43	1214.15			
Background	TD	56	1223.29	874.83	2500.50	12.56	.01*	.10
	ASD	56	971.58	789.86	2862.20			
Target	TD	56	1073.86	693.65	2932.20	1.23	.27	.01
	ASD	56	912.04	843.24	3175.80			
Face	TD	56	1876.96	872.62	3099.50	41.96	.00**	.28
	ASD	56	855.34	794.82	2967.70			
Target and face	TD	56	2950.82	789.26	3295.60	41.12	.00**	.27
	ASD	56	1739.53	1172.63	4047.70			

*TD* typically developing, *ASD* autism spectrum disorder

*p < .05: **p < .01

#### Eye-Gaze and Head-Turning Condition-2

In the Eye-Gaze and Head-Turning Condition-2, the study findings revealed significant differences between the two groups across all AOIs, with the exception of the Background AOI. Specifically, toddlers with ASD displayed significantly lower net dwell time in the Target AOI, Face AOI, and Target and Face AOI (F_t_ = 22.45, p = .00; F_f_ = 12.80, p = .01; F_tf_ = 35.62, p = .00) compared to the TD group. The effect sizes for these differences (n^2^ = .17, .10, .25) are interpreted as large, medium, and large effects respectively (Cohen, 1998). However, the net dwell time of children with ASD in the Body AOI was significantly higher than TD children (F_b_ = 14.20, p = .00) (See Table 2). This difference with an effect size of n^2^ = .11 is considered a medium effect (Cohen, 1998).

#### Eye-Gaze, Head-Turning, and Pointing Condition-3

In the Eye-Gaze, Head-Turning, and Pointing Condition-3, the findings showed that toddlers with ASD exhibited significantly lower net dwell time in the Target AOI, Face AOI, and Target and Face AOI (F_t_ = 5.07, p = .03; F_f_ = 20.08, p = .00; F_tf_ = 21.36, p = .00). The effect sizes for these differences (n^2^ = .04, .15, .16) are classified as small and large effects. In contrast, toddlers with ASD had significantly higher net dwell time in the Background AOI (F_b_ = 5.72, p = .02) with a small effect size (n^2^ = .05) (Cohen, 1998) (See Table 2).

#### Eye-Gaze, Head-Turning, Pointing, and Look! Condition-4

Finally, in the Eye-Gaze, Head-Turning, Pointing, and Look! Condition-4, study results indicated that toddlers with ASD displayed significantly lower net dwell time compared to their TD peers in three AOIs; the Background AOI, Face AOI, and Target and Face AOI (F_b_ = 12.56, p = .01; F_f_ = 41.96, p = .00; F_tf_ = 41.12, p = .00). The effect sizes for these differences (n^2^ = .10, .28, .27) are considered as medium and large effects (Cohen, 1998) (See Table 2).

### Intra-group Analysis

Children’s net dwell time across different AOIs Target AOI, Face AOI, and Target and Face AOI, Body AOI, Background AOI was examined for intra-group differences based on joint attention cue conditions using a two-way repeated measures ANOVA test. The dataset was divided based on group, gender, and a combination of both variables to assess differences between groups, gender, and their interactions. The joint attention condition was selected as the within-subjects variable, while group and gender were chosen as the between-subjects factors. Pairwise comparisons were conducted to determine whether the net dwell time of each AOI varied across joint attention conditions. The results of the ANOVA test are presented in Table 3A, B.

**Table 3:** Intra-group comparisons of net dwell time between conditions

(A)			
AOI	Contrast	Group	
	Conditions	ASD	TD
Body	E versus E + H	E = E + H	E = E + H
	E versus E + H + P	E < E + H + P**	E < E + H + P**
	E versus E + H + P + V	E < E + H + P + V*	E < E + H + P + V**
	E + H versus E + H + P	E + H < E + H + P*	E + H < E + H + P**
	E + H versus E + H + P + V	E + H < E + H + P + V*	E + H < E + H + P + V*
	E + H + P versus E + H + P + V	E + H + P = E + H + P + V	E + H + P = E + H + P + V
Background	E versus E + H	E = E + H	E = E + H
	E versus E + H + P	E > E + H + P*	E > E + H + P*
	E versus E + H + P + V	E > E + H + P + V**	E > E + H + P + V**
	E + H versus E + H + P	E + H = E + H + P	E + H = E + H + P
	E + H versus E + H + P + V	E + H > E + H + P + V*	E + H > E + H + P + V**
	E + H + P versus E + H + P + V	E + H + P = E + H + P + V	E + H + P > E + H + P + V*
Target	E versus E + H	E < E + H**	E < E + H**
	E versus E + H + P	E < E + H + P**	E < E + H + P**
	E versus E + H + P + V	E < E + H + P + V**	E < E + H + P + V**
	E + H versus E + H + P	E + H < E + H + P**	E + H = E + H + P
	E + H versus E + H + P + V	E + H = E + H + P + V	E + H = E + H + P + V
	E + H + P versus E + H + P + V	E + H + P = E + H + P + V	E + H + P > E + H + P + V*
Face	E versus E + H	E = E + H	E > E + H**
	E versus E + H + P	E = E + H + P	E > E + H + P**
	E versus E + H + P + V	E = E + H + P + V	E = E + H + P + V
	E + H versus E + H + P	E + H = E + H + P	E + H = E + H + P
	E + H versus E + H + P + V	E + H = E + H + P + V	E + H < E + H + P + V**
	E + H + P versus E + H + P + V	E + H + P = E + H + P + V	E + H + P < E + H + P + V**
Target & face	E versus E + H	E = E + H	E = E + H
	E versus E + H + P	E < E + H + P**	E = E + H + P
	E versus E + H + P + V	E < E + H + P + V**	E < E + H + P + V*
	E + H versus E + H + P	E + H < E + H + P*	E + H = E + H + P
	E + H versus E + H + P + V	E + H < E + H + P + V*	E + H < E + H + P + V**
	E + H + P versus E + H + P + V	E + H + P = E + H + P + V*	E + H + P < E + H + P + V*

(B)							
AOIs	Group	Condition	Mean	ss	F	p	Pairwise comparisons
Body AOI	TD (N = 56)	1	200.29	124.85	14.26	.00	1 = 2 (p = 1.00), 1 < 3 (p = .00**), 1 < 4 (p = .00**)
		2	245.52	159.19			2 = 1 (p = 1.00), 2 < 3 (p = .00**), 2 < 4 (p = .03*)
		3	466.17	345.66			3 > 1 (p = .00**), 3 > 2 (p = .01*), 3 = 4 (p = 1.00)
		4	493.77	373.59			4 > 1 (p = .01*), 4 > 2 (p = .03*), 4 = 3 (p = 1.00)
	ASD (N = 56)	1	327.48	297.21	7.98	.00	1 = 2 (p = .08), 1 < 3 (p = .00**), 1 < 4 (p = .01*)
		2	428.92	327.57			2 = 1 (p = .08), 2 < 3 (p = .4*), 2 < 4 (p = .03*)
		3	607.33	552.68			3 > 1 (p = .00**), 3 > 2 (p = .04*), 3 = 4 (p = 1.00)
		4	635.87	631.43			4 > 1 (p = .01*), 4 = 2 (p = .22), 4 = 3 (p = 1.00)
Background AOI	TD (N = 56)	1	1519.21	741.71	17.81	.00	1 = 2 (p = 1.00), 1 > 3 (p = .01*), 1 > 4 (p = .00**)
		2	1351.6	632.52			2 = 1 (p = 1.00), 2 > 3 (p = .02*), 2 > 4 (p = .00**)
		3	1029.29	552.74			3 < 1 (p = .01*), 3 < 2 (p = .02*), 3 > 4 (p = .02*)
		4	719.88	603.94			4 < 1 (p = .00**), 4 < 2 (p = .00**), 4 < 3 (p = .02*)
	ASD (N = 56)	1	1987.22	1031.59	8.66	.00	1 = 2 (p = .16), 1 > 3 (p = .01*), 1 > 4 (p = .00**)
		2	1642.91	936.93			2 = 1 (p = .16), 2 = 3 (p = .13), 2 > 4 (p = .03*)
		3	1352.2	846.04			3 < 1 (p = .01*), 3 = 2 (p = .13), 3 = 4 (p = 1.00)
		4	1223.29	874.83			4 < 1 (p = .00**), 4 < 2 (p = .03*), 4 = 3 (p = 1.00)
Target AIO	TD (N = 56)	1	467.90	371.91	43.21	.00	1 < 2 (p = .00**), 1 < 3 (p = .00**), 1 < 4 (p = .00**)
		2	1194.95	672.84			2 > 1 (p = .00**), 2 = 3 (p = .81), 2 = 4 (p = 1.00)
		3	1389.01	643.72			3 > 1 (p = .00**), 3 = 2 (p = .81), 3 > 4 (p = .03*)
		4	1073.86	693.65			4 > 1 (p = .00**), 4 = 2 (p = 1.00), 4 < 3 (p = .03*)
	ASD (N = 56)	1	248.69	272.93	23.40	.00	1 < 2 (p = .00**), 1 < 3 (p = .00**), 1 < 4 (p = .00*)
		2	666.34	494.15			2 > 1 (p = .00**), 2 < 3 (p = .00**), 2 = 4 (p = .10)
		3	1062.63	873.42			3 > 1 (p = .00**), 3 > 2 (p = .00**), 3 = 4 (p = .91)
		4	912.04	843.24			4 > 1 (p = .00**), 4 = 2 (p = .10), 4 = 3 (p = .91)
Face AOI	TD (N = 56)	1	1727.48	914.92	20.39	.00	1 > 2 (p = .00**), 1 > 3 (p = .00**), 1 = 4 (p = 1.00)
		2	1130.13	755.13			2 < 1 (p = .00**), 2 = 3 (p = 1.00), 2 < 4 (p = .00**)
		3	1171.01	643.13			3 < 1 (p = .00**), 3 = 2 (p = 1.00), 3 < 4 (p = .00**)
		4	1876.96	872.62			4 = 1 (p = 1.00), 4 > 2 (p = .00**), 4 > 3 (p = .00**)
	ASD (N = 56)	1	877.85	757.46	3.73	.02	1 = 2 (p = .05), 1 = 3 (p = .05), 1 = 4 (p = 1.00)
		2	666.21	609.60			2 = 1 (p = .05), 2 = 3 (p = 1.00), 2 = 4 (p = .24)
		3	638.09	615.33			3 = 1 (p = .05), 3 = 2 (p = 1.00), 3 = 4 (p = .20)
		4	855.34	794.82			4 = 1 (p = 1.00), 4 = 2 (p = .24), 4 = 3 (p = .20)
Target & face AOI	TD (N = 65)	1	2195.39	996.94	11.13	.00	1 = 2 (p = 1.00), 1 = 3 (p = .15), 1 < 4 (p = .00**)
		2	2325.08	933.66			2 = 1 (p = 1.00), 2 = 3 (p = .72), 2 < 4 (p = .00**)
		3	2560.03	772.56			3 = 1 (p = .15), 3 = 2 (p = .72), 3 < 4 (p = .02*)
		4	2950.82	789.26			4 > 1 (p = .00**), 4 > 2 (p = .00**), 4 > 3 (p = .02*)
	ASD (N = 65)	1	1126.55	809.33	7.99	.00	1 = 2 (p = .15), 1 < 3 (p = .00**), 1 < 4 (p = .00**)
		2	1330.76	826.13			2 = 1 (p = .15), 2 < 3 (p = .01*), 2 < 4 (p = .02*)
		3	1700.60	1157.45			3 > 1 (p = .01*), 3 > 2 (p = .01*), 3 = 4 (p = 1.00)
		4	1739.53	1172.63			4 > 1 (p = .00**) 4 > 2 (p = .02*), 4 = 3 (p = 1.00)

(A)* E* eye, *H* head, *P* point, *V* voice

*p < .05 two-tailed, **p < .01 two-tailed

(B) *TD* typically developing, *ASD* autism spectrum disorder, *AOI* area of interest, eye-gaze condition-1, eye-gaze and head-turning condition-2, eye-gaze, head-turning, and pointing condition-3, eye-gaze, head-turning, pointing, and “Look!” condition-4

*p < .05, **p < .01

#### Body AOI

In the Body AOI, the results indicated significant differences in net dwell time (as seen in Table 3A, B) between both study groups, toddlers with ASD and TD toddlers (F_TD_ = 14.26, p = .00; F_ASD_ = 7.98, p = .00; p < .05). A pairwise comparison analysis was conducted to identify the origins of these variations. The analysis indicated that the ASD group’s net dwell time in the Body AOI was higher in Conditions 4 and 3 than Condition 1. For toddlers with ASD, the net dwell time was also higher in Conditions 4 and 3 compared to Condition 2. Similarly, the TD toddlers exhibited a higher net dwell time in Conditions 4 and 3, compared to Conditions 2 and 1 (see Fig. 1A).

#### Background AOI

The ANOVA test revealed a significant difference in net dwell time depending on joint attention cue conditions (as seen in Table 3A, B) for both study groups, toddlers with ASD and TD toddlers (F_TD_ = 17.81, p = .00; F_ASD_ = 8.66, p = .00; p < .05). The pairwise analysis showed that the ASD group’s net dwell time was higher in Condition 1 compared to Conditions 3 and 4. In addition, while no significant difference was observed between Conditions 2 and 3, the ASD group’s net dwell time in Condition 2 was higher than in Condition 4. TD toddlers exhibited a similar pattern, with higher net dwell time in Conditions 1 and 2 compared to Conditions 3 and 4 (see Fig. 1B).

#### Target AOI

According to the ANOVA test, there was a significant difference in the net dwell time of study groups, toddlers with ASD and TD toddlers depending on the joint attention cue conditions in the Target AOI (F_TD_ = 43.21, p = .00; F_ASD_ = 23.40, p = .00; p < .05). As can be seen from Table 3A, B, the pairwise analysis revealed that toddlers with ASD spent significantly greater net dwell time on the Target AOIs in Conditions 4, 3, and 2 compared to Condition 1. Furthermore, the net dwell time of toddlers with ASD was also higher in Condition 3 than in Condition 2. However, there was no significant difference between Conditions 3 and 4 as well as Conditions 2 and 4. Similarly, TD toddlers showed greater net dwell time in Conditions 4, 3, and 2 compared to Condition 1. Their net dwell time was also significantly higher in Condition 3 than in Condition 4. Yet, there was no significant difference between Condition 2 and either Conditions 3 and 4 (see Fig. 1C).

#### Face AOI

In the Face AOI, findings indicated significant net dwell time differences (as seen in Table 3A, B) only in TD toddlers (F_TD_ = 20.39, p = .00; F_ASD_ = 3.73, p = .02; p < .05) based on joint attention cue conditions. The pairwise analysis revealed that TD toddlers spent significantly greater net dwell time on the Face AOI in Condition 1 compared to Conditions 2 and 3. The net dwell time of the TD toddlers was also higher in Condition 4 compared to Conditions 2 and 3 (see Fig. 1D). Although the p-value of F_ASD_ indicates a significant difference between conditions in the Face AOI for ASD toddlers, the pairwise analysis did not identify any significant difference between the conditions.

#### Target & Face AOI

In the Target and Face AOI, study findings revealed significant net dwell time differences based on joint attention cue conditions (as seen in Table 3A, B) for both toddlers with ASD and TD toddlers (F_TD_ = 11.13, p = .00; F_ASD_ = 7.99, p = .00; p < 0.05). As seen in Table 3A, B, the pairwise analysis indicated that the ASD had higher net dwell time in Condition 4 and Condition 3 compared to both Condition 2 and Condition 1. A similar trend were obtained in TD toddlers, who exhibited higher net dwell time in Condition 4 than in Conditions 3, 2, and 1 (see Fig. 1E).

#### Gender Differences

In addition to intra-group analyses comparing toddlers with ASD to TD toddlers, we also examined gender differences within each group. Between subject analysis revealed a significant gender difference between toddlers with ASD in the Face AOI (F = 7.95, p = .01). Specifically, male toddlers displayed a significantly lower net dwell time in the Face AOI than female toddlers. However, no gender differences were observed in any AOIs between the TD toddlers. Regarding the four joint attention conditions, male toddlers with ASD displayed significantly lower net dwell time in Condition 1 (t = − 2.15, p = .04), Condition 2 (t = − 2.95, p = .01), and Condition 3 (t = − 2.31, p = .02) than their female peers with ASD. No significant gender difference was found for toddlers with ASD in Condition 4 within the Face AOI (see Table 4).

**Table 4:** Intra-gender comparisons of net dwell time between conditions

AOI	Status	Conditions	Gender	B	Std. error	t	p	Partial eta squared
Face AOI	TD	1	Male	− 159.91	246.45	− .65	.52	.008
			Female	.00a				
		2	Male	118.67	203.56	.58	.56	.006
			Female	.00a				
		3	Male	− 97.17	173.41	− .56	.58	.006
			Female	.00a				
		4	Male	− 338.25	231.44	− 1.46	.15	.038
			Female	.00a				
Face AOI	ASD	1	Male	− 529.13	246.83	− 2.14	.04*	.078
			Female	.00a				
		2	Male	− 566.13	192.05	− 2.95	.00**	.139
			Female	.00a				
		3	Male	− 460.45	199.25	− 2.31	.02*	.090
			Female	0a				
		4	Male	− 487.292	261.527	− 1.863	.068	.060
			Female	.00a				

a. This parameter is set to zero because it is redundant

*TD* typically developing, *ASD* autism spectrum disorder

*p < .05: **p < .01

### Correlations Between Visual Attention and Clinical Information

Pearson correlation coefficient analysis was used to identify the clinical characteristics associated with the visual attention of both groups. Factors such as chronological and cognitive age, language and motor skills, and composite scores were included in the study. Table 5 displays the correlations between these clinical characteristics and visual attention measures for both groups. The study examined the net dwell time scores across five AOIs in four joint attention conditions. Several significant associations were found for both toddlers with ASD and TD toddlers, with a greater number of significant correlations observed in young children with ASD.

**Table 5:** Pearson’s correlations between net dwell time in four conditions and scores of clinical characteristics

Moderator variables (ASD vs TD)																				
AOI				Age				Language						Motor						
				Chronological		Cognitive		Composite		Expressive		Receptive		Composite			Fine		Gross	
				ASD	TD	ASD	TD	ASD	TD	ASD	TD	ASD	TD	ASD	TD		ASD	TD	ASD	TD
Body	Condition																			
	1	r		.09	− .08	− .10	− .10	− .09	− .07	− .04	− .06	− .05	− .07	.03	− .04		.04	− .13	.05	.01
		p		.51	.56	.46	.46	.50	.62	.79	.64	.70	.63	.81	.77		.76	.33	.70	.94
	2	r		.07	.04	− .14	− .02	− .25	.01	− .19	.17	− .18	.09	− .09	.09		.07	.05	.04	.02
		p		.61	.74	.32	.87	.06	.97	.16	.22	.19	.53	.53	.50		.63	.74	.78	.89
	3	r		.17	.23	.05	.21	− .10	.05	− .01	.18	− .03	.17	− .06	.11		.01	.25	− .06	.12
		p		.21	.09	.73	.12	.45	.74	.97	.18	.81	.22	.67	.44		.97	.07	.64	.38
	4	r		− .03	.08	− .17	.15	− .24	.19	− .15	.13	− .18	.12	− .15	.09		− .15	.26	− .13	.00
		p		.80	.54	.20	.28	.07	.15	.27	.33	.19	.38	.28	.50		.27	.05*	.34	.98
Background	Condition																			
	1	r		.41	.02	.27	− .03	.07	.20	.25	.03	.25	.01	− .14	.19		.19	.07	.13	.10
		p		.00**	.91	.04*	.80	.59	.14	.07	.85	.06	.95	.32	.17		.16	.59	.36	.47
	2	r		.18	.02	.26	.05	.06	.22	.14	.15	.08	.12	.00	.11		.13	.13	.01	.09
		p		.18	.86	.05	.72	.68	.10	.29	.26	.56	.38	.98	.42		.32	.33	.92	.51
	3	r		.03	− .06	− .15	− .12	− .13	− .04	− .07	− .12	− .08	− .21	.01	− .15		− .11	− .04	− .05	− .09
		p		.85	.65	.26	.36	.35	.77	.59	.38	.57	.12	.94	.28		.41	.75	.69	.51
	4	r		.05	.27	.03	.23	− .02	.04	.03	.17	.01	.25	.11	.05		.02	.34	.07	.15
		p		.70	.04*	.83	.08	.88	.79	.80	.21	.96	.06	.41	.73		.86	.01*	.59	.26
Target	Condition																			
	1		r	.13	− .03	.30	− .06	.29	.01	.25	− .02	.23	.10	.10	.06		.20	.01	.09	− .05
			p	.33	.84	.02*	.66	.03*	.94	.06	.88	.09	.47	.47	.65		.14	.91	.49	.70
	2		r	.26	.01	.40	− .06	.44	− .05	.45	− .10	.43	− .04	.12	− .04		.36	− .01	.29	− .10
			p	.05*	.92	.00**	.65	.00**	.74	.00**	.44	.00**	.76	.36	.77		.01*	.91	.03*	.48
	3		r	.19	.14	.42	.11	.46	− .14	.44	.13	.42	− .03	.10	− .06		.27	.16	.25	.00
			p	.16	.32	.00**	.40	.00**	.31	.00**	.33	.00**	.81	.47	.69		.05	.23	.07	.98
	4		r	.07	− .26	.23	− .26	.35	− .16	.26	− .37	.25	− .33	.12	− .14		.06	− .15	.02	− .28
			p	.59	.05*	.08	.06	.01*	.23	.05*	.01*	.07	.01*	.37	.30		.67	.25	.87	.04*
Face	Condition																			
	1		r	.00	− .05	.19	.01	.28	− .02	.24	− .03	.16	− .01	.17	− .09		.02	− .16	− .05	.11
			p	.98	.70	.17	.93	.04**	.91	.08	.84	.25	.93	.22	.49		.88	.24	.72	.40
	2		r	− .02	.02	.24	.15	.24	− .06	.17	.11	.17	.10	.15	.01		.10	− .02	.01	.07
			p	.87	.91	.07	.28	.08	.65	.21	.44	.22	.45	.26	.96		.48	.88	.93	.59
	3		r	.02	− .25	.32	− .18	.17	− .06	.10	− .12	.13	− .10	.05	− .06	.03		− .29	.01	− .11
			p	.88	.07	.01*	.18	.20	.68	.48	.38	.34	.45	.73	.65	.84		.03*	.96	.41
	4		r	.01	− .13	.23	− .05	.23	.03	.19	.11	.20	.10	.19	− .01	.12		− .20	.01	.04
			p	.94	.35	.09	.74	.09	.83	.16	.42	.15	.44	.16	.96	.38		.14	.93	.78
Target & face	Condition																			
	1		r	.04	− .06	.28	− .01	.36	− .01	.31	− .03	.22	.03	.19	− .06	.09		− .14	− .01	.08
			p	.76	.66	.04*	.94	.01*	.94	.02*	.81	.10	.85	.17	.64	.53		.30	.92	.53
	2		r	.14	.02	.42	.08	.44	− .08	.40	.01	.38	.05	.19	− .02	.28		− .03	.18	− .01
			p	.30	.87	.00**	.58	.00**	.55	.00**	.94	.00**	.70	.17	.86	.03*		.85	.18	.94
	3		r	.15	− .09	.49	− .06	.44	− .16	.39	.01	.39	− .11	.10	− .10	.22		− .11	.19	− .09
			p	.26	.50	.00**	.68	.00**	.23	.00**	.94	.00**	.41	.46	.48	.11		.43	.16	.51
	4		r	.04	− .37	.32	− .28	.40	− .11	.33	− .20	.32	− .17	.23	− .13	.14		− .36	.05	− .21
			p	.74	.01*	.02*	.04*	.00**	.41	.01*	.14	.02*	.21	.09	.33		.31	.01*	.73	.13

*TD* typically developing, *ASD* autism spectrum disorder, eye-gaze condition-1, eye-gaze and head-turning condition-2, eye-gaze, head-turning, and pointing condition-3, eye-gaze, head-turning, pointing, and “Look!” condition-4

*p < .05, **p < .01, r = .00–.30 weak, .30–.70 moderate, .70–1.00 strong

The study revealed significant correlations between net dwell time and clinical variables in the Target AOI, the Face AOI, and the Target and Face AOI, whereas the Body AOI demonstrated weaker correlations. Additionally, all significant correlations for toddlers with ASD were positive, in contrast to TD toddlers, where some correlations were negative. For example, composite language scores for toddlers with ASD positively correlated with the net dwell time in the Target AOI across all conditions.

The highest correlation with toddlers with ASD was associated with cognitive scores r = .49, p = .00 in Condition 3, following Conditions 2, 4, and 1, indicating that as toddlers with ASD improve in cognitive skills, they increasingly focus on the Target and Face AOI. Similar correlations were also found in the Target AOI. The second highest correlation with toddlers with ASD was related to both expressive (r = .45, p = .00) and receptive language scores (r = .43, p = .00) in Condition 2, showing that as toddlers with ASD improve in language skills, they focus more on the Target AOI. In addition, language scores of toddlers with ASD were also correlated with more visual attention in Target and Face AIO in all conditions. On the other hand, TD participants’ language scores did not show any significant correlation with the net dwell time across any AOIs in all conditions, with the exception of a negative correlation between expressive language skills and net dwell time of Target AOI in Condition 4 (r = − .37, p = .01) (Shown in Table 5). This pattern suggests that TD children with better expressive language skills were more likely to focus more on the Face AOI in Condition 4, likely due to the use of the verbal cue “Look” in the condition.

The study identified significant correlations between the net dwell time of toddlers with ASD in the Target AOI across Conditions 1, 2, and 3 and their cognitive age (r = .30, p = .02; r = .40, p = .00; r = .42, p = .00 respectively). In contrast, there was almost no significant correlation between the net dwell time of TD toddlers and their cognitive age, except for a small but significant correlation in the Target and Face AOI in Condition 4 (r = − .28, p = .04).

The study also revealed a limited correlation between the net dwell time scores of both toddlers with ASD and TD toddlers concerning motor skills. Of all the observed scores, the highest correlation was between the net dwell time of toddlers with ASD in the Target AOI during Condition 2 and their fine motor skills (r = .36, p = .01). Overall, language skills emerged as the most important clinical factor that significantly correlated with the net dwell time of toddlers with ASD, especially in the Target AOI. Further details can be found in Table 5.

## Discussion

The present study was designed to investigate the differences in visual attention between toddlers with ASD and TD toddlers under single and multiple joint attention cue conditions. Four distinct joint attention conditions, ranging from implicit to explicit cues, were developed to examine the attention cuing paradigm. The evidence from this study provides strong support for differences in visual attention under all joint attention conditions. Consistent with previous research, group differences were found across all four joint attention conditions. Findings from the study revealed that toddlers with ASD spent less net dwell time on the Face AOI and Target and Face AOI compared to TD toddlers across all joint attention conditions. More specifically, in these two AOIs, ASD toddlers spent approximately 50% less net dwell time compared to their TD peers.

The pattern of within group differences was also significant across joint attention conditions. Results revealed that toddlers with ASD demonstrated no difference in the net dwell time on the Face AOI across all four joint attention cue conditions. This study has clearly identified that the visual attention of toddlers with ASD on the Face AOI remained consistent, regardless of the joint attention cue condition presented. In other words, as the intensity of the joint attention cues increased, there was no observable change in the visual attention of toddlers with ASD directed towards the Face AOI. Another significant finding of the current study was that as the joint attention cues increased from Condition 1 to Condition 4, toddlers with ASD increasingly directed their visual attention towards the Target AOI rather than the Face AOI. While TD toddlers also exhibited increased attention to the Target AOI, they demonstrated a clear ability to shift their visual attention to the Face AOI in both Conditions 1 and 4.

These results are consistent with previous studies and suggest that eye gaze does not trigger attention shifts, indicating that young children with ASD did not find eye gaze to be a compelling attention cue (Congiu et al., 2016; Jones et al., 2014; Leekam et al., 1998; Nation & Penny, 2008; Thorup et al., 2016; Vivanti et al., 2017). On the other hand, we discovered that increasing joint attention cues with explicit pointing gesture and verbal cue “Look!” caused visual attention changes to a larger extent than eye gaze alone in toddlers with ASD on the Target AOI.

Moreover, the current study found that as the joint attention cues progressed from implicit to explicit joint attention conditions, the differences in visual attention between toddlers with ASD and TD toddlers became clearer, particularly in the Face AOI. TD toddlers displayed a longer net dwell time on the Face AOI in Condition 1, the Eye Gaze Condition, compared to Conditions 2 and 3. These results indicate that during Condition 1, TD toddlers displayed more visual attention on the model’s face, possibly searching for additional social cues.

Furthermore, in comparison to Conditions 2 and 3, TD toddlers exhibited a greater net dwell time on the Face AOI in Condition 4, which incorporates Eye-Gaze, Head-Turning, Pointing, and the verbal cue “Look!”. This finding was also expected, suggesting that the “Look!” cue predominantly directed the TD toddlers’ attention to the Face AOI. These results further support that TD toddlers’ higher net dwell time on the Face AOI might be a clear indicator of their synchronized auditory-visual attention.

On the other hand, compared to Conditions 3 and 4, toddlers with ASD spend more net dwell time on the Background AOI in Conditions 1 and 2. These results offered a critical understanding of how toddlers with ASD perceive social cues in their environment. In the current study, toddlers with ASD preferred to visually explore the Background AOI over any natural social stimuli, such as the presence of a model on the screen. Our hypothesis in the present study was that young children with ASD display inhibitory control difficulties in visual attention tasks. Supportive of this hypothesis, in a recent study, Akin-Bulbul and Ozdemir (2023) examined visual attention differences between toddlers with ASD, toddlers with developmental delay (DD), and TD toddlers during meaningful and nonmeaningful imitation tasks. Study results showed that young children with ASD showed reduced visual attention to the Movement and Face AOIs compared to TD and DD peers. The study’s key finding regarding visual attention was that toddlers with ASD showed a clear pattern of intensive visual attention on the External AOI compared to their TD and DD peers during meaningful and nonmeaningful actions on objects and vocal imitations. Overall, in the current study toddlers with ASD displayed a distinct pattern of visual attention on the Background AOI compared to other AOIs, whereas TD toddlers displayed a clear visual attention tendency on the Face AOI. During all joint attention conditions, the net dwell time of ASD toddlers was either very similar to or higher than the net dwell time of TD toddlers only in non-joint attention AOIs, including the Background and Body AOIs.

The present study replicates and extends the earlier research findings on decreased visual attention across joint attention conditions in toddlers with ASD by experimentally manipulating the joint attention cues. Over the past four decades, joint attention impairments in children with ASD have been primarily examined in observational studies (Charman et al., 1997; Dawson et al., 2004; Osterling & Dawson, 1994; Osterling et al., 2002; Wong & Kasari, 2012), whereas eye-tracking studies have recently been published to examine visual attention during joint attention bids in children with ASD (Billeci et al., 2016; Cilia et al., 2019). Observational studies raise questions regarding the emphasis on the absence versus the presence of frequency of joint attention skills and the lack of sensitivity to detect early joint attention impairments in ASD (Franchini et al, 2019). Examining visual attention as a quality indicator of joint attention, on the other hand, is essential for learning more about joint attention impairment in toddlers with ASD. Overall, the results of this study demonstrated that toddlers with ASD can be distinguished from TD toddlers by their visual attention to joint attention AOIs. From implicit to explicit joint attention cue conditions, we discovered that toddlers with ASD spent greater net dwell time in joint attention AOIs, with the exception of the Face AOI. Overall, current study findings clearly indicate that toddlers with ASD require more overt joint attention cues than TD toddlers to successfully respond to joint attention bids.

The second question in this research was to examine the correlations between the child’s characteristics and visual attention outcomes. The findings of this study showed that the cognitive scores of toddlers with ASD had positive and significant correlations with the net dwell time in the Target and Face AOI across all conditions, suggesting that toddlers with better cognitive scores tend to focus more on the Target and Face AOI and Target AOI. Language scores (both expressive and receptive language scores) were the second clinical score that revealed significant correlations with the net dwell time scores of toddlers with ASD in the Target and Face AOI and Target AOI. These results provide important insights into the role of cognitive and language characteristics of toddlers with ASD on their visual attention during joint attention conditions.

To the best of our knowledge, this is the first eye tracking study to show that toddlers with ASD, who exhibit greater visual attention in joint attention AOIs are more likely to display better early cognitive and language skills. These findings underscore the potential of eye tracking as an assessment methodology for identifying developmental strengths in toddlers with ASD and could inform targeted interventions to support their cognitive and language outcomes. Current study findings support previous behavioral research that links clinical characteristics of children with ASD and joint attention impairments. Over the past several decades, most research on joint attention has reported the correlations between children with ASD’s joint attention skills and their social, cognitive, adaptive, and language skills (Harrison et al., 2016; Mundy et al., 1994, 2009a, 2009b; Sano et al., 2021). Researchers reported significant correlations between children’s cognitive scores and joint attention skills (Harrison et al., 2016; Mundy et al., 1994; Sano et al., 2021). In a study, Leekam et al. (1998) showed that children with ASD with verbal mental age over 48 months were spontaneous gaze-followers compared to children in the low verbal mental age group.

Visual attention is one of the critical mechanisms of triadic joint attention, allowing young children to efficiently select the most relevant information while avoiding information overload. Thus, the findings from the current study are expected because joint attention plays a critical role in the development of communication skills and has been accepted as a transition to children’s first words (Bates et al., 1979; Braddock & Brady, 2016). In our perspective, joint attention exchanges lay a foundation for neurocognitive development because children learn to connect their attention with another’s visual, linguistic, emotional, and behavioral input. This foundation facilitates children’s triadic interactions where children communicate their thoughts about a third entity or individual to someone else (Mundy & Newel, 2007).

Finally, the analysis between subjects revealed a significant difference between genders only in toddlers with ASD in the Face AOI. Study findings suggest that male toddlers with ASD had lower net dwell time in the Face AOI compared to female toddlers in Conditions 1, 2, and 3. These sex differences in visual attention align with sex-typical patterns in literature (Harrop et al., 2018, 2019) indicating that females with ASD attend more to faces in socially lean conditions than males with ASD.

The current study has several limitations including the limited number of participants and a single TD comparison group. This study did not include a control group of children with language and cognitive delays that matched the chronological age and language equivalent of children with ASD. As a result, it’s challenging to determine whether the visual attention in joint attention conditions was more influenced by general cognitive and language differences between children with ASD and TD children rather than being specific to autism. Additionally, using a prerecorded joint attention stimulus is considered a relatively different experience than a face-to-face natural joint attention experience. Recent conceptual and empirical developments consistently indicate the need for investigations that allow the study of real-time social encounters in a truly interactive manner. This suggestion is based on the premise that social cognition is fundamentally different when we are in interaction with others rather than merely observing them. Nevertheless, eye-tracking technology has high accuracy in identifying attentional orientation (e.g., Jones et al., 2014; Nation & Penny, 2008) and visual engagement with the target of another person’s gaze (Falck-Ytter et al., 2015; Freeth et al., 2010; Riby et al., 2013). Furthermore, future research can investigate visual attention during joint attention bids utilizing an improved ecological paradigm in which the child’s visual attention can be investigated in real-life situations rather than in computer-based joint attention scenes. High-precision, non-invasive sensor-based remote monitoring systems can be employed to achieve natural and realistic examinations of visual attention in children by tracking their eye movements in their natural environment.
